# The burden of headache in China: validation of diagnostic questionnaire for a population-based survey

**DOI:** 10.1007/s10194-011-0336-2

**Published:** 2011-03-31

**Authors:** Sheng-Yuan Yu, Xiu-Tang Cao, Gang Zhao, Xiao-Su Yang, Xiang-Yang Qiao, Yan-Nan Fang, Jia-Chun Feng, Ruo-Zhuo Liu, Timothy J. Steiner

**Affiliations:** 1 Department of Neurology, Chinese PLA General Hospital, Fuxing Road 28, Haidian District, Beijing, 100853 China; 2 Department of Health and Economics, Chinese PLA General Hospital, Beijing, China; 3 Department of Neurology, The Fourth Military Medical University, Xian, Shaanxi Province China; 4 Department of Neurology, Xiaya Hospital of Centre-south University, Changsha, Hunan Province China; 5 Department of Neurology, Affiliated Huashan Hospital of Fudan University, Shanghai, China; 6 Department of Neurology, The First Affiliated Hospital of Sun Yat-sen University, Guangzhou, Guangdong Province China; 7 Department of Neurology, The First Hospital of Jilin University, Changchun, Jilin Province China; 8 Department of Neuroscience, Norwegian University of Science and Technology, Trondheim, Norway

**Keywords:** Epidemiology, Questionnaire, Headache, Migraine, Tension-type headache, Burden of headache

## Abstract

The objective of this study was to test the validity, in the Chinese population, of the *Lifting The Burden* diagnostic questionnaire for the purpose of a population-based survey of the burden of headache in China. From all regions of China, a population-based sample of 417 respondents had completed the structured questionnaire in a door-to-door survey conducted by neurologists from local hospitals calling unannounced. They were contacted for re-interview by telephone by headache specialists who were unaware of the questionnaire diagnoses. A screening question ascertained whether headache had occurred in the last year. If they had, the specialists applied their expertise and ICHD-II diagnostic criteria to make independent diagnoses which, as the gold standard, were later compared with the questionnaire diagnoses. There were 18 refusals; 399 interviews were conducted in 202 women and 197 men aged 18–65 years (mean age 44.4 ± 12.6 years). In comparison to the specialists’ diagnoses, the sensitivity, specificity, positive predictive value, negative predictive value and Cohen’s kappa (95% CI) of the questionnaire for the diagnosis of migraine were 0.83, 0.99, 0.83, 0.99 and 0.82 (0.71–0.93), respectively; for the diagnosis of tension-type headache (TTH), they were 0.51, 0.99, 0.86, 0.92 and 0.59 (0.46–0.72), respectively. In conclusion, the questionnaire was accurate and reliable in diagnosing migraine (agreement level excellent), less so, but adequate, for TTH (sensitivity relatively low, false negative rate relatively high and agreement level fair to good). The non-specific features of TTH do not lend themselves well to diagnosis by questionnaire.

## Introduction

Primary headache disorders, in particular migraine (MIG) and tension-type headache (TTH), are very common throughout the world, and a cause of widespread and substantial disability. According to the World Health Organization (WHO), MIG on its own is the 19th highest cause of disability worldwide [1]. Evidence of this came from surveys of the prevalence of MIG in many countries, but not all. Since this WHO finding, studies have embraced more and more countries, but not yet China, whose population is close to one-fifth of that of the entire world. An epidemiological study conducted in the southeast area of China in 1986 reported the prevalence of MIG as 0.987% [2, 3]. This is unfeasibly much lower than prevalences elsewhere, including nearby Japan [4] and Taiwan [5], and is suspect because the survey predated the International Classification of Headache Disorders (ICHD) [6] and therefore did not use the accepted diagnostic criteria of ICHD. Moreover, it did not assess burden, and there has been no other estimation of the burden of headache in the mainland of China.

A population-based door-to-door survey of the prevalence and the burden of primary headache disorders in the Peoples’ Republic of China was initiated by *Lifting The Burden*: the Global Campaign against Headache [7]. The study reported here aimed to validate the diagnostic questionnaire and the survey method.

## Methods

### Ethics

The study protocol was approved by the Chinese Ministry of Health and the ethics committee of the Chinese PLA General Hospital, Beijing.

### Questionnaire

The structured questionnaire was adapted from the English version developed by *Lifting The Burden* for population-based studies. As in similar surveys supported by *Lifting The Burden*, the questionnaire had three parts: (a) personal and socio-demographic data; (b) diagnostic questions related to MIG, TTH and chronic daily headache (CDH) disorders including medication-overuse headache (MOH); (c) enquiry into headache-related burden. Additionally, height, weight, abdominal circumference and blood pressure were included.

The address and telephone number of respondents were recorded for purposes of verification, if required. The personal demographic data included age, gender, nationality and marital status. Socioeconomic status of respondents was assessed from educational level, occupation and total household income per year.

The diagnostic questions began with a screening question “Have you had a headache in the last year not related to flu, hangover, cold or head injury?”, as recommended by earlier studies. The respondents who answered “no” were asked no further questions except those related to quality-of-life; those who answered “yes” were next asked about frequency (headache days per month or year). Respondents reporting headache on ≥15 days per month were questioned on usage of medications over the previous 3 months in order to identify MOH.

Further diagnostic questions were based on ICHD-II criteria [8] and aimed to identify MIG and TTH. Respondents who might have more than one type of headache were instructed to focus on the most bothersome headache. The purpose of this was to ensure, as far as possible, that they gave their responses with only one headache type in mind. The questions included frequency, duration, quality, site and intensity of headache, accompanying symptoms (nausea, vomiting, photophobia and phonophobia) and the impact of physical activity on headache. The questionnaire neglected trigeminal autonomic cephalalgias and secondary headaches, since these are infrequent and not significantly contributory to population headache burden.

A set of questions enquiring into point prevalence were commenced with the screening question “Did you have headache yesterday?”. The respondents who answered “yes” were asked about the duration and intensity of this headache and its effect on ability to perform daily activities.

The enquiry into burden consisted of five parts: (a) utilization of health care; (b) payments made, for diagnosis and/or treatment of headache, in the previous year; (c) headache-attributed lost time (from work, from home chores and socially), using the HALT index [9]; (d) willingness to pay (WTP) in future for putatively effective treatment; (e) impact of headache on quality-of-life, using the WHOQoL-8 question set (applied also to a control sub-sample without headache).

The questionnaire was translated into Chinese. In accordance with the *Lifting The Burden* translation protocol for hybrid documents [10], the Chinese version was first evaluated by the principal investigators (one from each of the six administrative regions of China), who were fluent in both languages, and then assessed for comprehensibility by 20 outpatients in the neurology departments of the hospitals where these principal investigators were working.

### Survey

The full survey, in all six regions of China, was carried out door-to-door by neurologists employed by local hospitals near to where the respondents lived. At a prior meeting at the lead centre in Beijing, at least one interviewer from each city was instructed in the methods of the survey and in ICHD-II; he or she then returned and trained other members of his/her team. Also prior to the main study, a pilot study surveyed 21 families in each region to test the efficiency of this epidemiological method.

To produce a population-based respondent sample, random-sampling software was developed by a statistician (X-T C), according to the EPI method established by WHO [11]. The method is summarized as follows. China has 27 provinces and 4 directly administered cities, which were merged into 24 units in a sampling frame based on population; from these 24 units, 35 cities or districts were selected for sampling and, from each of these, 20–30 towns or streets in proportion to the population. One village or community was randomly selected from each town or street, and one household from each village or community. The family in that household, and the families in the seven households occurring consecutively along a direction randomly chosen by the surveyors upon arrival at the first family, provided the survey sample. All members of each family were listed by age, and one adult (aged 18–65 years) was chosen as the respondent according to a random sample form.

The main survey included 5,041 households at which the interviewers called unannounced.

### Validation

After the survey, a sub-sample of respondents with or without headache were selected randomly from all respondents and asked to undergo second interviews. Each of these was conducted by telephone by one of the six principal investigators who was unaware at the time of the questionnaire diagnosis. These interviews determined whether the respondents had headache and, if so, made a diagnosis according to ICHD-II criteria [8]. Respondents reporting more than one type of headache were again asked to concentrate on the most bothersome headache.

The diagnoses made in these interviews were regarded as the gold standard, whereas the questionnaire diagnostic possibilities were limited to MIG, TTH, MOH or unclassified headache, the principal investigators applied their neurological expertise to make whatever diagnoses were appropriate.

### Statistics

Respondents’ data were processed in EpiData 2.1a and transferred into SAS 9.1.3 for statistical analysis. Sensitivity, specificity and positive (PPV) and negative predictive values (NPV) were calculated for the questionnaire diagnoses of MIG and TTH against the gold standard. Cohen’s kappa (*κ*) was calculated for the agreement between diagnoses. Guidelines suggest that values of *κ* above 75% indicate excellent agreement, values between 75 and 40% indicate good to fair agreement and those below 40% show poor agreement [12].

A 5% level of significance and 95% confidence intervals (CI) were used.

## Results

From the 5,041 households of the main survey, 491 respondents were selected for the sub-sample, of whom 417 were contacted; the other 74 were not available by telephone on either of two attempts. Interviews were completed in 399 of these (202 women and 197 men, mean age 44.4 ± 12.6 years, 131 from urban and 268 from rural areas), with 18 (4.3%) refusing whilst confirming that questionnaires had been finished in the main study.

The overall response rate was, therefore, 81.3%. Table 1 shows that there were response-rate differences between the different regions [the rate being especially low (55.6%) in the western region], between urban (78.4%) and rural (82.7%) areas, and between the majority Chinese Han (83.0%) and ethnic minority people (61.5%).

**Table 1:** Response rates to headache specialists’ telephone interview

	Responded, *n*	Refused, *n*	Failed contact, *n*	Response rate (%)
Region				
Northeast	36	2	9	76.6
North	80	3	4	92.0
Southeast	95	0	3	96.9
South	55	0	13	80.9
Central	83	5	13	82.2
Western	50	8	32	55.6
Habitation				
Urban	131	6	30	78.4
Rural area	268	12	44	82.7
Ethnicity				
Han	375	16	61	83.0
Minority	24	2	13	61.5
Total	399	18	74	81.3

In the main survey, headache in the previous year was reported by 84 (21.1%) of the 399 respondents, of whom 2 indicated more than one headache type; 315 (78.9%) had no headache. Headache on ≥15 days per month (CDH) was reported by 4 (1.0%) of the 399 respondents. According to the questionnaire diagnoses, 30 (7.5%) of the 399 had MIG and 35 (8.8%) had TTH. The headaches of the other 13 respondents, and those of the two with more than one headache type, were not classifiable according to the questionnaire.

At re-interview by the headache specialists, 91 (22.8%) of the 399 respondents reported headache in the previous year, a discordance of 7 (1.8%). Two (2.4%) were diagnosed as having more than one type of headache, these being the same as the two identified by questionnaire. (In Table 2, these are shown as unclassified headache.) Four (1.0%) of the 399 were diagnosed as CDH, 28 (7.0%) as MIG and 56 (14.0%) as TTH. Table 2 compares diagnoses by headache specialists and questionnaire.

**Table 2:** Agreement between questionnaire and headache specialist diagnoses

Questionnaire	Specialist					
	MIG	TTH	CDH	No headache	Unclassified	Total
MIG	24	2	0	4	0	30
TTH	1	30	1	3	0	35
CDH	0	0	4	0	0	4
No headache	3	12	0	300	0	315
Unclassified	0	12	0	1	2	15
Total	28	56	5	308	2	399

*MIG* migraine, *TTH* tension-type headache, *CDH* chronic daily headache

The sensitivity, specificity, PPV and NPV of the questionnaire for MIG and TTH were calculated (Table 3). These statistical indices demonstrated that the questionnaire was accurate and reliable in diagnosing migraine (sensitivity 0.83, specificity 0.99, *κ* = 0.82), but, for TTH, sensitivity was relatively low (0.51); despite a very high specificity (0.99), *κ* (0.59) indicated only fair to good agreement for TTH [12].

**Table 3:** Statistical indices of questionnaire diagnostic performance

	MIG	TTH
Sensitivity (95% CI)	0.833 (0.7968–0.8699)	0.51 (0.4594–0.5575)
Specificity (95% CI)	0.99 (0.9751–0.9978)	0.99 (0.9735–0.9971)
Coincidence (95% CI)	0.97 (0.9596–0.9903)	0.91 (0.8874–0.9422)
False negative rate	0.17	0.49
False positive rate	0.01	0.01
Youden index (95% CI)	0.82 (0.6859–0.9537)	0.49 (0.3656–0.6220)
PPV (95% CI)	0.83 (0.7000–0.9667)	0.86 (0.7412–0.9731)
NPV (95% CI)	0.99 (0.9747–0.9982)	0.92 (0.8925–0.9481)
*κ* (95% CI)	0.82 (0.7095–0.9301)	0.59 (0.4629–0.7242)

*MIG* migraine, *TTH* tension-type headache, *CI* confidence interval, *PPV* positive predictive value, *NPV* negative predictive value, *κ* Cohen’s kappa

## Discussion

In order to validate a headache screening and diagnostic questionnaire for a population-based burden of headache survey in the Peoples’ Republic of China, 491 respondents sampled randomly from 5,041 who had participated in the survey were re-interviewed by headache specialists by telephone. The specialists applied their expertise and ICHD-II criteria to make independent diagnoses of MIG, TTH, MOH or other primary or secondary headache disorder to be used as the gold standard against which questionnaire diagnoses were compared.

ICHD-II makes a distinction between definite migraine (dMIG) and probable migraine (pMIG) and between definite TTH (dTTH) and probable TTH (pTTH). In this study, we made these distinctions at first instance, although it should be recognized that, in epidemiological surveys, no cases are strictly “definite” by ICHD-II criteria since the last criterion (exclusion of other possible causes) cannot be applied. In practice, this is not a problem because other possible causes are uncommon. When all other criteria for migraine were met in the questionnaire survey, the diagnosis was dMIG; if one only was not met, we next applied the criteria for TTH and, when these were met, the diagnosis was dTTH; otherwise, the diagnosis was pMIG. Finally, when none of these applied, but one only of the criteria for TTH was unmet, the diagnosis was pTTH; otherwise, the case was listed as unclassified headache. Prior to this process, the few cases of CDH were identified and those with MOH set aside, with that diagnosis. The diagnoses by specialists followed normal rules, except that, because the interviews were by telephone, they also could not reliably exclude other possible causes.

For comparisons between questionnaire and specialist diagnoses, we then combined definite and probable cases of MIG and of TTH. The argument in support of this is as follows: all cases of dMIG and all cases of pMIG (being episodic headache that does not meet criteria for TTH) are more probably migraine than other type of headache, likewise for dTTH and pTTH [13]. In epidemiological studies, this approach is pragmatic and practical: a “probable” diagnosis has a purpose in clinical management, but it serves none as a separate category in population studies.

The study found high or very high sensitivity, specificity, PPV and NPV for MIG, with *κ* = 0.82 indicating excellent agreement [12] (Table 3). For MIG, therefore, the *Lifting The Burden* questionnaire in its Chinese-language version is accurate and reliable for the purpose of prevalence surveys in China, comparing favourably with other diagnostic questionnaires for this disorder [13–23]. The same questionnaire translated into Russian language and used in a Russian population performed not quite so well when applied by lay interviewers (sensitivity 0.77, specificity 0.82, *κ* = 0.58) [13].

For TTH, specificity was very high (0.99) but sensitivity (0.51) was low and false negative rate high (Table 3). As a result, *κ* = 0.59 indicated only fair to good agreement. Other questionnaires for the diagnosis of TTH are few. Rasmussen’s questionnaire performed less well (specificity 0.96, sensitivity 0.43) [18]. The Russian-language version of the *Lifting The Burden* questionnaire was of lower specificity (0.91) but more sensitive (0.64), with *κ* = 0.56 again indicating only fair to good agreement [13]. Kukava’s [14] Georgian-language instrument, applying multiple questionnaires in sequence for MIG, TTH and cluster headache, achieved still lower specificity (0.86) but higher sensitivity (0.79) for TTH. Thus, all questionnaires struggle, especially for sensitivity, with TTH. The non-specific features of TTH do not lend themselves well to diagnosis by questionnaire, and it appears difficult to control the false negative rate in TTH diagnosis. Furthermore, questionnaires that do not focus on MIG only but embrace more than one potential diagnosis generally achieve lower agreement levels. For example, Hagen et al. [19] differentiated between MIG, non-migraine headache and chronic headache with *κ* = 0.59, 0.43 and 0.44, respectively. The questionnaire of Rasmussen et al. [18] covered MIG and episodic and chronic TTH, with *κ* = 0.43, 0.30 and 0.24.

It is questionable whether, for example through further training of the interviewers, sensitivity for TTH can be improved. The experience in Russia, where lay interviewers were employed [13], suggests this is possible to some degree but at a cost in specificity. There are factors beyond control. The diagnostic process is bound to rely upon ICHD-II criteria, which were not designed for and may not be well adapted to epidemiological research. The key problem is the nature of TTH and, as stated above, its lack of specific features; it is easy to see how this might promote specificity to the disadvantage of sensitivity since any mention of any such feature pushes the diagnosis away from TTH. In this regard, the specific features of photophobia and phonophobia are, in particular, difficult to explain to respondents.

Our study had advantages and some limitations. Refusal of re-interview by 18 selected participants may have introduced bias but, if so, it was unlikely to be substantial. The 399 interviews conducted by the headache specialists included many screening negatively for headache, allowing detection of questionnaire false negatives. But, because they were performed by telephone, neurological or other examination could not be performed; in this way, the “gold standard” was defective. Since accurate headache diagnosis depends largely on a well-taken history, and other causes of headache are uncommon in comparison with the prevalences of MIG and TTH, any detriment consequent upon this was also unlikely to be substantial.

In conclusion, our results indicate that the *Lifting The Burden* diagnostic questionnaire, in Chinese, is fit for the purpose of a population-based burden of headache study in China, being sensitive and specific for MIG and, whilst imperfect, as adequate as any other for TTH.
